# Continuous Glucose Monitoring Enables the Detection of Losses in Infusion Set Actuation (LISAs)

**DOI:** 10.3390/s17010161

**Published:** 2017-01-15

**Authors:** Daniel P. Howsmon, Faye Cameron, Nihat Baysal, Trang T. Ly, Gregory P. Forlenza, David M. Maahs, Bruce A. Buckingham, Juergen Hahn, B. Wayne Bequette

**Affiliations:** 1 Department of Chemical & Biological Engineering, Rensselaer Polytechnic Institute, Troy, NY 12180, USA; howsmd@rpi.edu (D.P.H.); fmccamer@gmail.com (F.C.); baysan@rpi.edu (N.B.); hahnj@rpi.edu (J.H.); 2 Stanford University School of Medicine, Stanford, CA 94305, USA; tly@insulet.com (T.T.L.); buckingham@stanford.edu (B.A.B.); 3 Barbara Davis Center for Childhood Diabetes, University of Colorado Denver, Aurora, CO 80045, USA; gregory.forlenza@ucdenver.edu (G.P.F.); dmaahs@stanford.edu (D.M.M.); 4 Department of Biomedical Engineering, Rensselaer Polytechnic Institute, Troy, NY 12180, USA

**Keywords:** type 1 diabetes, fault detection, continuous subcutaneous insulin infusion, sensor-augmented pump

## Abstract

Reliable continuous glucose monitoring (CGM) enables a variety of advanced technology for the treatment of type 1 diabetes. In addition to artificial pancreas algorithms that use CGM to automate continuous subcutaneous insulin infusion (CSII), CGM can also inform fault detection algorithms that alert patients to problems in CGM or CSII. Losses in infusion set actuation (LISAs) can adversely affect clinical outcomes, resulting in hyperglycemia due to impaired insulin delivery. Prolonged hyperglycemia may lead to diabetic ketoacidosis—a serious metabolic complication in type 1 diabetes. Therefore, an algorithm for the detection of LISAs based on CGM and CSII signals was developed to improve patient safety. The LISA detection algorithm is trained retrospectively on data from 62 infusion set insertions from 20 patients. The algorithm collects glucose and insulin data, and computes relevant fault metrics over two different sliding windows; an alarm sounds when these fault metrics are exceeded. With the chosen algorithm parameters, the LISA detection strategy achieved a sensitivity of 71.8% and issued 0.28 false positives per day on the training data. Validation on two independent data sets confirmed that similar performance is seen on data that was not used for training. The developed algorithm is able to effectively alert patients to possible infusion set failures in open-loop scenarios, with limited evidence of its extension to closed-loop scenarios.

## 1. Introduction

Improvement in care for patients living with type 1 diabetes is currently tied to emerging external medical device technology, including continuous glucose monitors and insulin infusion pumps, seeking movement towards closed-loop artificial pancreas systems [1,2]. Since a goal of these devices is to reduce adverse events due to glucose excursions, artificial pancreas systems are often designed with patient safety rather than tight control as the highest priority. However, artificial pancreas systems still require patient involvement when changing sensors, choosing infusion set sites, and diagnosing potential problems. One major potential problem with any artificial pancreas system is the loss of the infusion set’s ability to actuate the system. Faulty actuation occurs for a number of reasons, including dislodged catheters, insulin leakage or lipohypertrophy at the infusion set site, and mechanical failure of the infusion set. Additional hazards associated with continuous subcutaneous insulin infusion are presented in [3,4]. Alarming a patient when a faulty infusion set is detected has the potential to mitigate or prevent the resulting hyperglycemic glucose excursions.

**Figure 1:**
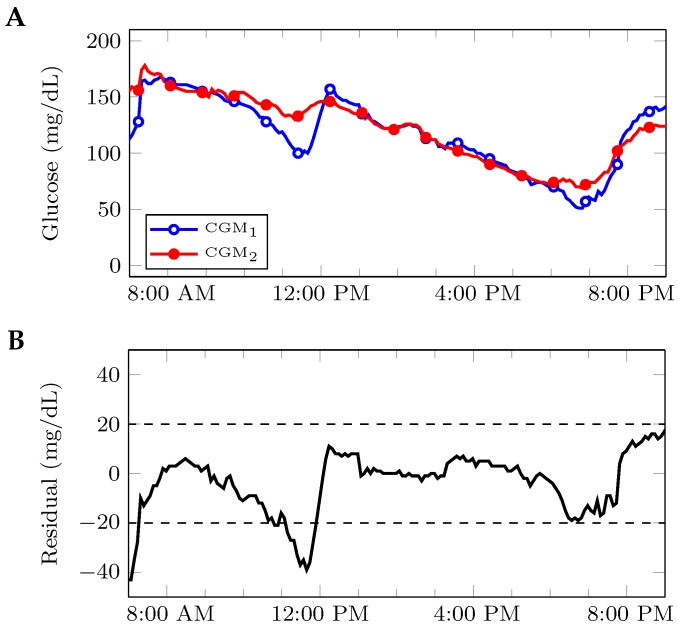
Illustration of continuous glucose monitor (CGM) sensor fault detection based on hardware redundancy. In this example, (**A**) a patient wears two CGM sensors at different locations, and (**B**) these signals are compared to generate a residual. A potential fault detection scheme based on hardware redundancy would analyze the residual for fault signatures; in this case, a simple threshold at ±20 mg/dL was used.

Fault detection strategies can be first classified into hardware- or analytically-redundant approaches. Hardware redundancy involves incorporating extra hardware for the sole purpose of detecting a particular fault. In the case of pressure-induced sensor attenuations (PISAs) [5], two continuous glucose monitors (CGMs) can be worn, and PISAs can be detected by directly comparing the outputs of these two sensors. Figure 1 illustrates how such an approach can be developed. Analytically-redundant approaches instead compare the data with a human’s understanding of that data. The interested reader is referred to [6] for an overview of fault detection systems based on analytical redundancy. In the context of infusion set faults, analytical redundancy is usually incorporated by either comparing the CGM signal with a model-derived glucose signal or analyzing characteristics of input (administered insulin) and output (glucose) signals.

Despite their promise to improve the safety of both open-loop and closed-loop insulin infusion strategies, there have only been a few strategies to detect losses in infusion set actuation (LISAs) proposed in the scientific literature, and even fewer evaluated on patient data. Kovács et al. transformed the Bergman Minimal Model (BMM) into an input-affine, linear parameter-varying space, and infusion set faults were detected via an unknown input filter [7]; however, the methodology was not evaluated on real patient data. A robust control strategy based on the Hovorka Model was formulated by Vega-Hernandez et al. [8,9], but again, the strategy was not evaluated on real patient data. The BMM was also used in an Unscented Kalman Filter framework and applied retrospectively to data from an intravenous glucose tolerance test [10]. A classifier based on multivariate statistical analysis was trained on simulated data from the UVa/Padova simulator [11,12]. A fault detection scheme requiring CGM and carbohydrate intake data based on modal interval analysis was applied to a modified BMM [13], and the fault detection algorithm was tested against the UVa/Padova simulator. Another approach [14,15] identified black-box state-space models, and these models were again evaluated on in silico data from the UVa/Padova simulator. A comparison of three different strategies was given by Baysal et al. [16], and the algorithms were finally evaluated on patient data. Cescon et al. [17] also evaluated infusion set failure algorithms on clinical data as opposed to simulated faults, but tried to anticipate—as opposed to detect—failures. Furthermore, these authors assumed that the patient will respond to every alarm by changing the infusion set, and they report a sensitivity and specificity of 55% and 66%, respectively.

Incomplete knowledge about the various causes of LISAs and their probabilities of occurrence creates much uncertainty around modeling sources of LISAs necessary in simulation studies. One of the main advantages of this work is the evaluation of the proposed LISA detection algorithm on substantial amounts of clinical data rather than simulated scenarios. Furthermore, the simplicity of the algorithm eases integration with artificial pancreas systems, requiring negligible computational resources.

## 2. Materials and Methods

This section defines the criteria for determining an infusion set failure, and follows with a discussion of the data and training procedure used in algorithm development. Illustrations are provided to demonstrate the core concepts of the proposed approach.

### 2.1. Notation

In the derivations that follow, *k* represents time in minutes, LW represents the length of the long window, SW represents the length of the short window, ${\bar{x}}_{i|j}$ represents the average of quantity *x* in a window from time *j* to time *i*, and $\Delta x_{k}$ represents the quantity $x_{k}-x_{k-1}$.

### 2.2. Algorithm Development

Insufficient insulin administration leads to elevated glucose levels. However, patients differ in their level of control, and fault detection algorithms should be robust to this inter-patient variability. Furthermore, patients may improve or degrade their level of control over time, leading to intra-patient variability. To account for the different levels of control and the possibility that an individual’s control may change over time, the fault detection algorithm makes use of the average glucose reading over a long time horizon, ${\bar{\mathrm{CGM}}}_{k|k-LW}$. This long-term CGM average provides a patient-specific baseline level of control. The average glucose in a shorter time horizon, ${\bar{\mathrm{CGM}}}_{k|k-SW}$, is used to capture only the most recent history of a patient’s glucose levels, and describes his/her current level of control. If ${\bar{\mathrm{CGM}}}_{k|k-SW}$ is greater than ${\bar{\mathrm{CGM}}}_{k|k-LW}$, the area between these two curves ${\mathrm{AUC}}_{k}$ is accumulated in the glucose fault metric (GFM). However, if ${\bar{\mathrm{CGM}}}_{k|k-SW}$ falls below ${\bar{\mathrm{CGM}}}_{k|k-LW}$, the GFM resets to a value of 0. Formally, the glucose metric can be described by:

(1) $$\begin{array}{cc} {\mathrm{AUC}}_{k} & =\left({\bar{\mathrm{CGM}}}_{k|k-SW}-{\bar{\mathrm{CGM}}}_{k|k-LW}\right)\cdot \Delta t_{k}\\ {\mathrm{GFM}}_{k} & =\left\{\begin{array}{cc} {\mathrm{GFM}}_{k-1}+{\mathrm{AUC}}_{k} & \mathrm{if}\;\;\frac{{\bar{\mathrm{CGM}}}_{k|k-SW}}{{\bar{\mathrm{CGM}}}_{k|k-LW}}>1\\ 0 & \mathrm{otherwise} \end{array}\right. \end{array}$$

**Figure 2:**
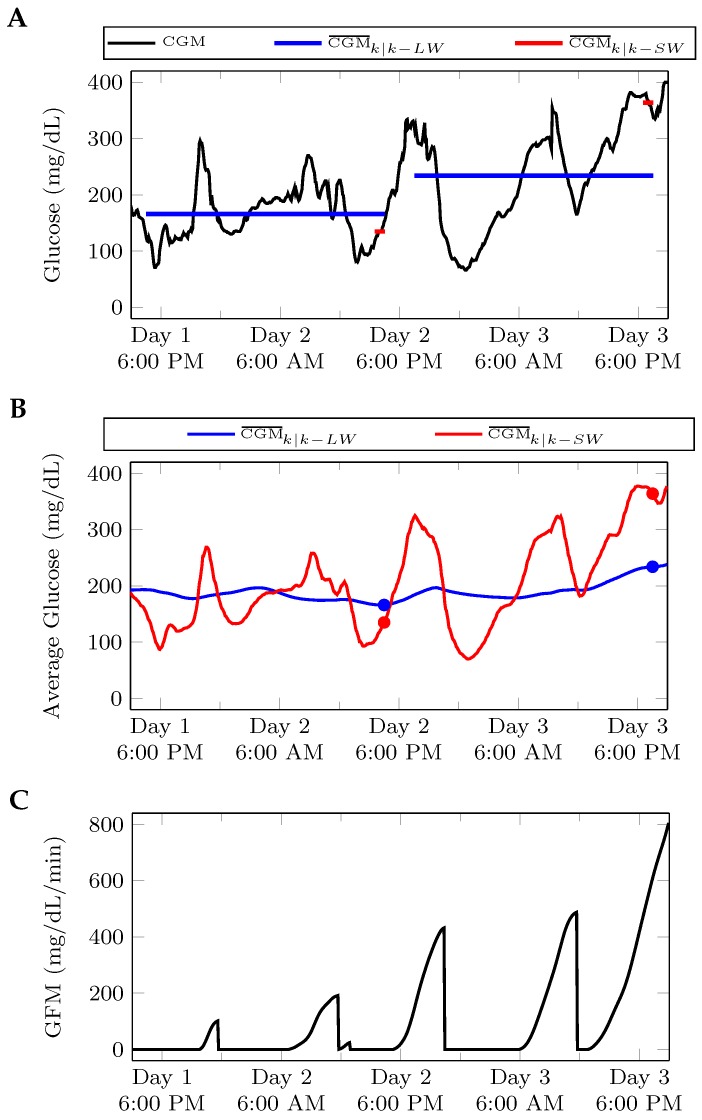
Illustration of the glucose fault metric (GFM) calculation for LW=24 h and SW=1 h. (**A**) The length of the horizontal lines corresponding to ${\bar{\mathrm{CGM}}}_{k|k-LW}$ and ${\bar{\mathrm{CGM}}}_{k|k-SW}$ indicate the length of time of long window (LW) and short window (SW), respectively; (**B**) averages are computed for each new data point. The marked points correspond to the horizontal bars in the top figure; (**C**) when ${\bar{\mathrm{CGM}}}_{k|k-SW}<{\bar{\mathrm{CGM}}}_{k|k-LW}$, GFM=0. However, when ${\bar{\mathrm{CGM}}}_{k|k-SW}>{\bar{\mathrm{CGM}}}_{k|k-LW}$, the area between these two curves accumulates in GFM.

To eliminate false positives, an alarm is only issued if the number and slope of glucose readings in the short time window are above certain thresholds. An illustration of the calculation of the GFM is presented in Figure 2.

Elevated glucose levels alone are not indicative of an infusion set failure. Indeed, some glucose excursions are definitely caused by meals. However, many patients do not choose to use the meal bolus calculators provided on their insulin infusion pumps, and some artificial pancreas algorithms do not require meal information for successful operation [18,19]. In an effort to make this algorithm as widely applicable as possible, meal information is not incorporated, but an additional insulin fault metric (IFM) is proposed. Insulin-on-board is a well-established metric in the diabetes community that describes a pharmacodynamic bound on the current plasma insulin levels [20]. Insulin-on-board can be described by passing the administered insulin through a first-order filter. However, since insulin must first travel through the subcutaneous compartment before it can affect a patient’s blood glucose levels, the administered insulin is passed through a second-order filter to obtain the plasma insulin estimate (PIE). The PIE then estimates the amount of active insulin in the blood, rather than the total amount of insulin injected in the body. PIE at time *k* is obtained from the linear system.

(2) $$\begin{array}{cc} \left[\begin{array}{c} x_{k+1}^{1}\\ x_{k+1}^{2} \end{array}\right] & =\left[\begin{array}{cc} 0.98 & 0.02\\ 0 & 0.98 \end{array}\right]\left[\begin{array}{c} x_{k}^{1}\\ x_{k}^{2} \end{array}\right]+\left[\begin{array}{c} 0\\ 1 \end{array}\right]{\mathrm{Insulin}}_{k}\\ {\mathrm{PIE}}_{k} & =\left[\begin{array}{cc} 1 & 0 \end{array}\right]\left[\begin{array}{c} x_{k}^{1}\\ x_{k}^{2} \end{array}\right] \end{array}$$

A one-minute sample time is used here, since insulin boluses and basal changes can occur at shorter time scales than the five minutes between most commercial CGM measurements. The variable ${\mathrm{Insulin}}_{k}$ is calculated by summing the total bolus insulin and the current basal rate in units per minute. This approach [21] and similar approaches [22] have been used in artificial pancreas systems. The average PIE in the short window ${\bar{\mathrm{PIE}}}_{k|k-SW}$ is compared to that in the long window ${\bar{\mathrm{PIE}}}_{k|k-LW}$. Since individual insulin requirements vary, this comparison is normalized by ${\bar{\mathrm{PIE}}}_{k|k-LW}$. Then, IFM is given by:

(3) $${\mathrm{IFM}}_{k}=\frac{{\bar{\mathrm{PIE}}}_{k|k-SW}-{\bar{\mathrm{PIE}}}_{k|k-LW}}{{\bar{\mathrm{PIE}}}_{k|k-LW}}=\frac{{\bar{\mathrm{PIE}}}_{k|k-SW}}{{\bar{\mathrm{PIE}}}_{k|k-LW}}-1$$

**Figure 3:**
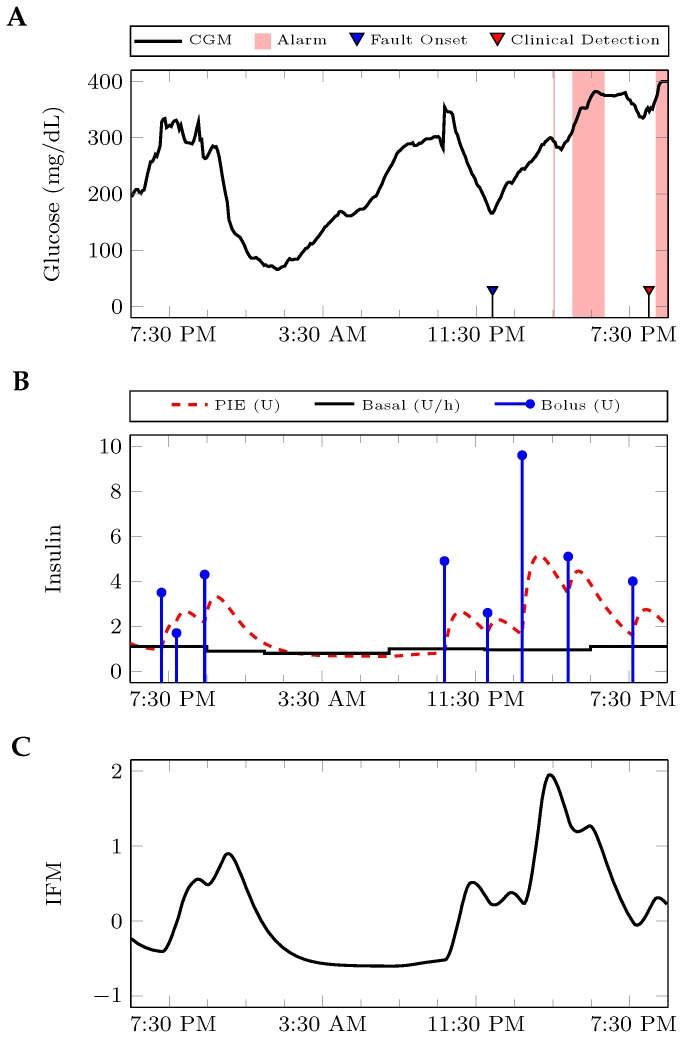
Illustration of the insulin fault metric (IFM) calculation for LW=24 h and SW=1 h. (**A**) The patient’s glucose level is given over time. Time points with retrospective alarms are shaded in red; (**B**) the basal and bolus insulin administration is passed to a second-order filter to determine the plasma insulin estimate (PIE); (**C**) the IFM calculated from the given insulin profile.

An example illustrating the function of the IFM is presented in Figure 3.

With the GFM and IFM defined, it is now possible to derive criteria for issuing an infusion set failure alarm that is independent of a patient’s specific level of control and insulin requirements. Thresholds are placed on the allowable GFM, IFM, and CGM slope in the short window. Exceeding all three of these thresholds simultaneously signals an alarm.

### 2.3. Training and Validation Data

Three different data sets were used to evaluate the performance of the LISA detection algorithm. The data set T1 used to train the system was taken from a study investigating the effect of infusing insulin in lipohypertrophic sites [23]. Failure dynamics were independent of the lipohypertrophy status of the infusion site [23]; therefore, all data were combined for training. Once the parameter set was determined from the training set, this algorithm was tested on completely independent data sets from one study V1 investigating Teflon versus steel infusion sets [24] and a different study V2 investigating the use of hyaluronidase for influencing the subcutaneous insulin pharmacokinetics [25]. Since hyaluronidase alters insulin pharmacokinetics, only the control group in V2 (no hyaluronidase administered) was used for validation. Testing on data independent from the training set ensures that the developed algorithm generalizes well to new patient data, and is not simply fit to the specific data used in algorithm generation. Additional details of the different data sets are described in Table 1.

### 2.4. Performance Evaluation

All data sets were obtained from studies that clinically detected infusion set failures; however, the exact time of these infusion set failures is not known, since these failures were not induced. Therefore, the start of the failure was selected retrospectively by a team of engineers and clinicians, and was generally determined to coincide with the most previous trough in the CGM data, as done previously [16]. Although this uncertainty in the true start time of a fault is a limitation of this approach, this drawback was deemed favorable to creating faults in a simulation environment that makes considerable assumptions as to glucose–insulin dynamics and the dynamics of LISAs. The uncertainty in the true start of an infusion set fault coupled with the more realistic scenario of a patient responding to an alarm event rather than each serial data point prompted the evaluation of the algorithm based on alarm events rather than individual data points. Sensitivity was calculated based on whether or not an alarm sounded between the estimated start time and the clinically detected fault, and false positives were also evaluated on an event basis. Since true negatives and therefore specificity cannot be determined in this framework, parameters in the LISA algorithm were chosen to maximize sensitivity and minimize the number of false positives per day in a pseudo-receiver operating characteristic curve (pROC) curve. This analysis based on alarm events follows previous performance evaluation procedures used for the detection of infusion set failures [16].

## 3. Results and Discussion

**Figure 4:**
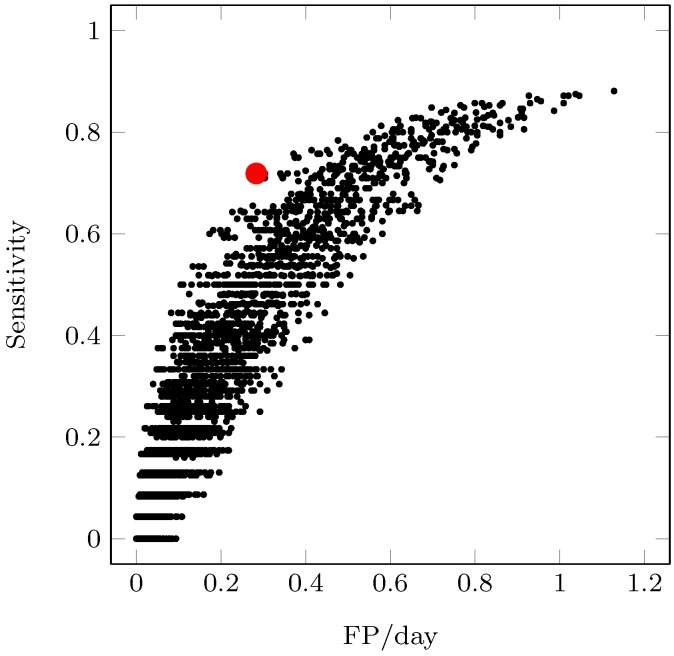
The pROC generated from the performance of different algorithm parameter sets on T1. The marker for the chosen parameter set is enlarged and highlighted in red.

A parameter set consisting of the (1) long time window length; (2) short time window length; (3) GFM threshold; (4) IFM threshold; and (5) glucose slope threshold was determined via the pROC curve. The parameter set with metrics closest to the upper left corner was chosen for future analysis. Figure 4 displays the pROC curve, and Table 2 lists the parameter ranges investigated and the chosen parameter values. The “⋯” in Table 2 indicate a continuation of the previous step size until the end value is reached. All combinations of the parameters listed in Table 2 were investigated and plotted in Figure 4. With the chosen values, the algorithm was able to achieve a 71.8% sensitivity with 0.28 false positives per day. It is important to note that the LISA detection algorithm sent alerts at fairly high glucose levels (Table 1), indicating that the LISA detection algorithm will not completely prevent hyperglycemia following an infusion set failure. However, since these failures were detected earlier by the algorithm than by the patients, LISA-induced hyperglycemia will likely be mitigated if the LISA detection algorithm were implemented in real-time.

Once suitable parameters were obtained from analysis of the training set, the LISA detection algorithm was validated against V1 and V2. Failure characteristics were independent of the infusion set material; therefore, all infusion sets in V1 were evaluated as a single group. Table 1 summarizes the performance of the algorithm in these validation studies. Since the validation results closely resembled the training results, the algorithm generalizes to new data sets well.

Additionally, the validation results of V1 obtained the same sensitivity with fewer false positives per day than the algorithms tested by Baysal et al. [16] that were trained on this data. These previous algorithms are described briefly as: (1) the multivariate statistical analysis (MSA) decomposes fault metrics via principal components and compares the magnitude of the first principal component and the glucose slope in a two hour window to determine a fault; (2) the model-based analysis (MBA) uses an interactive multiple model approach to simulate one case when insulin is fully ineffective and one case where insulin is fully effective, and these predictions are combined in a Bayesian probabilistic environment; and (3) the Threshold approach simply alarms only when the glucose level reaches 305 mg/dL, and will not issue a subsequent alarm for at least six hours to reduce false positives. The comparison results are highlighted in Table 3. One important observation in any of the algorithms tested on data set V1 is that the sensitivity is 73%. One explanation for this result is the fact that faults are quickly addressed by the clinical staff in these studies rather than allowing the possibly faulty conditions to continue for a longer portion of time. Indeed, the missed faults had an average duration or 4.9 h, whereas the detected faults had an average duration of 10.6 h. Presumably, these missed detections would be eventually detected, but patients cannot be subjected to these clinically unsafe conditions for ethical reasons.

One inherent assumption of the LISA detection algorithm is the pharmacokinetic profile of insulin. When hyaluronidase was administered to decrease the time to peak insulin effect, the LISA detection algorithm only achieved a sensitivity of 44.4% and issued 0.33 FP/day, differing markedly from the performance when hyaluronidase was not administered. Therefore, this algorithm should be retrained with a new insulin pharmacokinetic profile if the injected insulin differs significantly from the assumed profile.

Although the algorithm issued fewer false positives than previous approaches, patients may experience alarm fatigue with the current system. However, the largest source of false positives was attributed to under-bolused meals, since—for all patients that provided meal information—80.2% of false positives occurred within two hours of a meal (the median time since the previous meal was 63 min). Furthermore, the median glucose reading when a false alarm occurred was 292 mg/dL across all data sets. Thus, the false positives may improve clinical outcomes by alerting patients to scenarios of hyperglycemia requiring more insulin delivery. Furthermore, incorporating meal information is an important future direction to reduce false positives for patients who wish to provide this information to a fault detection algorithm.

Although this algorithm was developed for use in the presence of meals, the algorithm performs similarly when the fault occurs during the night time where only basal insulin is administered. Across all data sets, there were three cases of infusion set failure at night, and all three of these cases were correctly detected by the algorithm with similar mean glucose and time to detection as reported in Table 1. These results are a promising indication that the LISA detection algorithm can correctly identify faults overnight; however, algorithms specific to detecting faults in the absence of meals may perform better in this scenario [14].

The algorithm was developed on open-loop data to improve the current level of care; however, future commercial artificial pancreas devices will need to focus on fault detection and intra-patient variability more than in current clinical trials. Therefore, the algorithm was then evaluated on ten infusion sets from a closed-loop study at a diabetes camp [26]. The algorithm successfully detected the two infusion sets in the study and issued 0.32 false positives per day. These results suggest that the algorithm may extend into closed-loop scenarios with similar performance. Since this closed-loop data set is rather limited, ongoing studies are under investigation as to the performance of this LISA detection algorithm in online, closed-loop scenarios.

## 4. Conclusions

Diabetes technology seeks to improve clinical outcomes and reduce patient burden. Although this increase in a patient’s quality of life is advantageous under everyday scenarios, it can also lead to a decreased awareness toward adverse events as systems become more automated. Therefore, future devices will need to incorporate fault detection strategies to a greater degree than the current state-of-the-art. The algorithm developed herein was able to detect infusion set failures in open-loop scenarios, and may extend to closed-loop scenarios with similar performance. Detecting adverse actuator faults will improve the safety and performance of artificial pancreas devices by alerting patients to check—and possibly replace—their infusion sets.

## Figures and Tables

**Table 1:** Data set characteristics and algorithm performance. FP: false positive.

	T1	V1	V2
Reference	[23]	[24]	[25]
Number of patients	20	18	13
Number of infusion sets	62	49	22
Total patient days	352.7	275.7	106.9
Number of infusion set failures	23	15	10
Algorithm Sensitivity	71.8%	73.3%	71.4%
Algorithm FP/day	0.28	0.27	0.28
Algorithm Median Minutes to Detect	262	210	280
Algorithm Glucose at Detection (mg/dL)	289	300	264

**Table 2:** Parameter ranges tested in algorithm development and the selected values from the pseudo-receiver-operating characteristic (pROC) analysis.

Parameter Name	Units	Parameter Range	Selection
LW	h	{6,12,⋯,36}	24
SW	h	{5/60,0.5,1,⋯,6}	1
GFM threshold	(mg/dL)·min	{50,75,⋯,400}	100
IFM threshold	unitless	{0,0.05,⋯,1}	0.4
Glucose Slope threshold	(mg/dL)·min	{0,0.05,⋯,2}	0.3

**Table 3:** Data set characteristics and algorithm performance. MBA: model-based analysis; MSA: multivariate statistical analysis.

Algorithm Name	LISA	MBA	MSA	Threshold
Reference	—	[16]	[16]	[16]
Sensitivity	73%	73%	73%	73%
FP/day	0.27	0.43	0.36	0.33
Median Minutes to Detect	210	181	240	225
Detection Glucose (mg/dL)	300	277	315	313
Validation Results?	✓	✗	✗	✗

## Abbreviations

The following abbreviations are used in this manuscript:

CGM	continuous glucose monitoring
CSII	continuous subcutaneous insulin infusion
LISA	loss in infusion set actuation
PISA	pressure-induced sensor attenuation
BMM	Bergman minimal model
GFM	glucose fault metric
PIE	plasma insulin estimate
IFM	insulin fault metric
FP	false positive
pROC	pseudo-receiver operating characteristic curve
MSA	multivariate statistical analysis
MBA	model-based analysis
